# Better cardiac care – the patient experience – a qualitative study

**DOI:** 10.1186/s12939-023-01931-5

**Published:** 2023-06-28

**Authors:** Warren Jennings, Sonya Egert, Celestine Fisher, Sonia Renouf, Vivian Bryce, Sean Grugan, William Wang, Deborah Askew

**Affiliations:** 1 Southern Queensland Centre of Excellence in Aboriginal and Torres Strait Islander Primary Health Care, Metro South Hospital and Health Service, 37 Wirraway Parade, Inala, Qld, 4077 Australia; 2 General Practice Clinical Unit, The University of Queensland, Women’s Hospital, Level 8, Health Sciences Building, Royal Brisbane, Brisbane, Qld 4029 Australia; 3 Princess Alexandra Hospital, Queensland Health, 199 Ipswich Road, Woolloongabba, Qld 4102 Australia; 4 Faculty of Medicine, The University of Queensland, 199 Ipswich Road, Woolloongabba, Qld 4102 Australia

**Keywords:** Aboriginal and Torres Strait Islander people, Indigenous, Cultural competence, Equity, Health access, Relationality, Racism, Health worker, Yarning, Qualitative, Cardiac care.

## Abstract

**Background:**

In 2015, a Brisbane tertiary hospital’s cardiac unit implemented a new model of multidisciplinary care (Better Cardiac Care (BCC)) for Aboriginal and Torres Strait Islander patients. Since then, clinical indicators for Aboriginal and Torres Strait Islander cardiac patients have improved, but the recipients’ voices have not been heard. This research aimed to determine the acceptability and appropriateness, features of value, and opportunities for improvements in this model of care, from the perspective of patients and their family members.

**Methods:**

This descriptive qualitative study employed a narrative methodology. BCC Health Workers contacted prospective participants; with consent, interested individuals were then contacted by the Aboriginal Research Officer (RO) who arranged yarning sessions and consent. Family members were also invited to share their stories of their loved ones’ hospitalisation. Two researchers conducted the interviews, using a yarning approach. Inductive narrative analysis, informed by Aboriginal and Torres Strait Islander ways of Being, Knowing, and Doing, focused on enabling participants’ stories to be heard and understood from their perspectives.

**Results:**

Relationality was at the heart of the BCC model of care, particularly between patients and Aboriginal and Torres Strait Islander staff. The relationality included a responsibility for holistic care, extending beyond hospital discharge, although support and handover for family members required improvement. The Aboriginal and Torres Strait Islander staff understood the contextual and structural challenges faced by participants, including the disempowerment and racism experienced in healthcare. This understanding was shared with the BCC team who, in turn, protected, advocated for, and holistically supported participants through their cardiac health journeys.

**Conclusions:**

Empowering (and employing) Aboriginal and Torres Strait Islander staff, and relating to patients as people, enabled BCC to meet Aboriginal and Torres Strait Islander patient’s needs and improve outcomes. The wider health system and health academia could benefit from exploring and valuing Aboriginal and Torres Strait Islander discourses of relationality.

## Background

The life expectancy gap between Aboriginal and Torres Strait Islander people and non-Indigenous Australians, driven by social determinants of health, is one of the most pressing social justice challenges facing Australian society [1]. Although many social determinants are beyond the influence of the health system, disparity in healthcare access is one which the health system must own and address.

Cardiac disease contributes 19% of the gap in total disease burden between Aboriginal and Torres Strait Islander people and their non-Indigenous counterparts [2]. However, Aboriginal and Torres Strait Islander people are less likely to receive timely diagnosis of cardiac disease and less likely to receive guideline-based therapy for acute coronary syndrome [2]. This inequitable disparity was first documented in 2003-04, when Aboriginal and Torres Strait Islander people were 40% less likely to receive angiography or percutaneous coronary interventions and 20% less likely to undergo coronary artery bypass grafting than non-Indigenous Australians [3]. This gap has reduced since then, but its continuing existence remains unacceptable.

In response, national policy recommendations to improve hospital cardiac care for Aboriginal and Torres Strait Islander people were developed. These included improving the coordination, cultural competence, and representation of Aboriginal and Torres Strait Islander health staff within the cardiac space [4]. Within this context, in 2015 the Princess Alexandra Hospital (PAH) in Brisbane, Australia, implemented the Better Cardiac Care (BCC) program to improve the quality of care for Aboriginal and Torres Strait Islander people admitted under cardiac and cardiothoracic teams. BCC, developed in consultation with Aboriginal and Torres Strait Islander and non-Indigenous stakeholders, included a multidisciplinary team consisting of clinical nurse consultants (CNCs), pharmacists, administrative officer, cardiologists, and an Aboriginal and Torres Strait Islander Hospital Liaison Officer (HLO) evolving to Health Workers (HWs) over the course of this study [5]. The BCC team delivered changed health communication strategies, medication support, discharge planning and follow-up, and day-to-day contact and support [6]. One year after its introduction, all-cause unplanned cardiac readmissions had reduced by 21%, and unplanned acute myocardial infarction readmission had reduced by 21%, compared to historical controls [6].

While these improvements in clinical outcomes are promising, the experiences of recipients of this new model of care have not been explored and therefore understanding why the model of care has made a difference is lacking. Previous research investigating the experiences of Aboriginal and Torres Strait Islander patients admitted to cardiology units have identified a range of unmet needs, interpersonal experiences that negatively impacted on their hospital experience [7, 8] and institutional racism all contributing to higher rates of discharging against medical advice [9], which is associated with increased morbidity, mortality and health system costs [10, 11]. Here, we aimed to (i) understand the experiences of Aboriginal and Torres Strait Islander patients and their family members of BCC, particularly in relation to patient satisfaction, perceptions of cultural safety, health care engagement, patient empowerment and health literacy, (ii) understand how this may have differed to previous care, and iii) identify opportunities for improvement of this model of care.

## Methods

### Researcher positionality

The research team included three qualitative researchers independent from the PAH (SE, WJ, DA), and five BCC team members – a cardiologist (WW), two CNCs (VB and SG) and an Aboriginal and Torres Strait Islander Hospital Liaison Officer (SR) and Health Worker (CF). The BCC team members helped to plan the study and participant recruitment but had no role in data collection nor analysis. SE, a research officer (RO), is a Noonuccal Goenpul woman of the Quandamooka Nation of Southeast Queensland. DA (senior researcher) and WJ (a General Practitioner clinician researcher) are non-Indigenous researchers who had undertaken cultural awareness training but understood that cultural awareness, including cultural humility, is a lifelong journey. SE and DA had attended training in Yarning methods [12] conducted by Prof Dawn Bessarab, the originator of this research approach. When the study commenced, SE, WJ and DA worked at the Southern Queensland Centre of Excellence in Aboriginal and Torres Strait Islander Primary Health Care (SQCoE), a state-government run health service providing comprehensive primary health care to the Aboriginal and Torres Strait Islander people of Inala and surrounding suburbs.

### Qualitative approach and research paradigm

This qualitative study used an Aboriginal and Torres Strait Islander research approach in which we aimed to recognise Aboriginal and Torres Strait Islander world views, knowledges and realities, privilege Aboriginal and Torres Strait Islander voices and experiences and support self-determination and sovereignty [13]. The Aboriginal member of the SQCoE research team (SE) improved the trustworthiness and safety-checking of the reporting, analysis and interpretation of story providers’ voices. The respectful and reciprocal approach to helped to disrupt the historical dominance of Eurocentric approaches in Aboriginal and Torres Strait Islander health research was challenged[14].

### Ethical considerations and aboriginal and Torres Strait Islander governance

The study was conducted according to the guidelines of the Declaration of Helsinki, the National Health and Medical Research Council (NHMRC) National Statement on Ethical Research in Humans and the NHMRC Guidelines for Ethical Conduct in Aboriginal and Torres Strait Islander Health Research [15, 16]. Ethics approval was granted by the Metro South Health Human Research Ethics Committee (HREC/2019/QMS/50,743).

To ensure Aboriginal and Torres Strait Islander governance and to enact Indigenous Data Sovereignty, we sought approval from the Inala Community Jury for Aboriginal and Torres Strait Islander Health Research [17] to conduct the research and publish its outcomes.

### Recruitment

Aboriginal and Torres Strait Islander people have been story tellers since time immemorial. The telling of stories ensured the teachings of their ways of knowing, being and doing were passed from one generation to the next and the role of “story teller” is synonymous with the role of teacher. In recognition of this, and borrowing from Wain et al. (2016), we use the terms “story provider” and “story collector” to demonstrate the disruption to the normative power structurers of interviewer and passive interviewee [18]. Critical case sampling [19] was used to identify story providers who were perceived to have particularly rich and informative stories of relevance to the topic and aims of this research. Aboriginal and Torres Strait Islander people who had been admitted to the PAH under the BCC team, aged 18 years and older, were eligible to participate in this study. We aimed to have a broad cross-section of story providers, across age, sex, length of stay, and metropolitan or rural/regional residence.

On request of the Community Jury, we amended the study protocol to enable the collection of stories from family members.

To respect cultural and ethical protocols, a multi-staged approach to recruitment was implemented. First, the BCC team generated a list of Aboriginal and Torres Strait Islander patients admitted in the previous six months, then removed those considered to be too ill, emotionally distressed, or cognitively impaired to participate. Next, the list was anonymised, each person’s reason for admission, length of stay, and date of discharge was added and the list provided to SE, DA and WJ to select potential story providers. The BCC HLO or HW then phoned the selected individuals to discuss the research. With permission, the contact details of interested individuals were passed on to SE for recruitment conversations.

When first making contact with participants, SE positioned herself, relative to the potential story providers, by providing her full name (her family is well known in the Aboriginal and Torres Strait Islander communities of Inala and beyond) and where she worked [20]. She reminded the potential story provider of the processes that had been followed to gain their contact details, and reassured them that their privacy and confidentiality were respected. For those who agreed to participate, a time and place for the interview was negotiated.

When relevant, story providers identified a key family member who had been involved in their cardiac journey during the research yarn. If one was identified and agreed to participate, a suitable time and place was identified for the research yarn.

### Story collection

Yarning, a rigorous and credible research method for data gathering when researching with Aboriginal and Torres Strait Islander people, was used for all interviews [12]. Yarning involves “an informal and relaxed discussion through which both the researcher and the participant journey together visiting places and topics of interest relevant to the research” [12]p38. It does not impose a preferred structure or sequence for data collection, but rather, recognises the data in the stories and allows for a free-flowing narrative that includes what matters to each story provider. To ensure the cultural safety of all story providers, SE participated in all but one of the yarns together with WJ or DA. On this occasion, SE contacted the participant to notify them that it would be two non-Indigenous story collectors, and provide the participant the opportunity to reschedule the interview if they wished.

Establishment of a relationships between story providers and story collectors is fundamental to the yarning process. The foundations for a trusting relationship had been previously laid through the initial phone conversation that SE had had with each story provider, and were strengthened through social yarning at the commencement of the story collection [12, 18]. Consent to participate and to audio-record the Yarns was obtained (written in face to face Yarns, verbal consent for telephone or videocall Yarns) prior to any data collection.

Story providers were asked to recount their experiences of being admitted to the PAH for cardiac care, and to compare their experience of being admitted under the BCC team to previous admissions at the PAH or other hospitals. Research yarns with story providers living in Brisbane were face-to-face and via telephone or videocall with those living distant to Brisbane. Yarns lasted between 30 and 60 min. Story providers were given an AUD25 gift voucher in recognition of their contribution to the research. Audiorecordings were transcribed professionally and deidentified by SE prior to analysis. Story collection occurred from 22nd November 2019 to 9th September 2021.

### Data analysis

In keeping with Aboriginal and Torres Strait Islander methodology, an inductive narrative approach was used for data analysis whereby SE. WJ and DA interpreted, contextualised and compared the collected stories with each other [21]. Thus, the significance of each story provider’s experience was understood from their own perspective and context. Following the Emden 1998 approach [22], transcripts were converted into narratives with a clear chronological structure. These narratives were read and reread and sub-plots within each story were identified, and then compared and contrasted (i.e. across-case narrative analysis) [23]. The qualitative research team met regularly throughout this process to discuss and interpret findings and, informed by Karen Martin’s theoretical framework, considered how the BCC program embodied Aboriginal and Torres Strait Islander ways of knowing, being and doing [24]. NVivo [25] was used to manage data, and an audit trail of the research processes and analysis was kept to maximise reliability. Data analysis and reporting was informed by the COREQ guidelines for qualitative research [26], and by the Aboriginal and Torres Strait Islander Quality Appraisal Tool (QAT) [27].

### Impact of the COVID-19 pandemic

The COVID-19 pandemic disrupted recruitment for over 12 months as both the SQCoE and PAH pivoted away from research to focus on patient care. By early 2021, restrictions had sufficiently eased and recruitment and data collection recommenced. Travel restrictions and concerns about physical proximity with medically vulnerable individuals remained leading to telephone and videocall yarns being offered.

## Results

### Participants

A total of 16 patients, all Aboriginal, were interviewed (11 male and five female), plus two family members (both female, one was a Torres Strait Islander person, and one was non-Indigenous). All participants were from south-eastern Queensland: seven were from regional centres, included an inner-regional discrete Aboriginal community, and 11 were from Greater Brisbane (inclusive of the Brisbane, Ipswich and Logan city council areas). Participants had a mix of ages, from the elderly to those with younger children, and a mix of cardiac conditions, with acute coronary presentations, recurrent disease processes (e.g. cardiac failure) and long-term disease processes (e.g. rheumatic heart disease).

### Ways of being – power and relationality

The *relationships* that patient-participants had with the BCC team, particularly with the HLOs and HWs, were highly valued, and enabled the BCC team to care holistically for patient-participants as people, rather than just as malfunctioning hearts. There was a social, caring relationality between the BCC team and patient-participants which disrupted the usual hospital power structures, empowered the BCC team to advocate for patient-participants, and enabled the patient-participants to leave hospital with the medication and follow-up care they needed. The connection patient-participants felt to the BCC team was often anchored to the HLOs and HWs due to shared cultural knowledges and contemporary lived experiences. One patient-participant explained how she could be herself, relaxed and connected with the BCC HLO, as opposed to the ‘whitefella’ voice and communication style she adopted with non-Indigenous people.

*Because I can talk to her (BCC HLO) in a way that I know that she will understand, whereas if I talk that way to a non-Indigenous person, or a non-Indigenous person who hasn’t had a lot of experience with Aboriginal and Torres Strait Islander people then … I’d have to speak with my whitefella voice … when I was working, and I’ve worked for some really big reputable organisations, where I’ve been on the phone and I’ve had to speak, if I’m speaking to a non-Indigenous person, I would speak to them in a way that they could understand … it’s just relating to people, I guess whereas if I had a blackfella come in, a client, I could just talk to them like I was talking to my cousin … I can relate … and same lingo and everything like that ... so it does make a difference … we do live in a white man’s society really. But then there’s people who you’re around who you can be just yourself, and normal … (P02A)*

All participants described how the BCC team fulfilled their relational responsibilities to the patient, not only during their inpatient period but also before and after the admission with telephone calls to check on the individual’s health and wellbeing. Patient-participants recounted ‘popping in’ to the BCC offices for a visit after discharge, and some recounted their own reciprocity of giving back to the team through gifts of artwork or volunteered time. Patient-participants felt connected to the BCC team and felt treated as family, as they themselves treated the BCC team as family, as the following extract describes.

*When your family turn up and they [BCC] say we’ll get going – no, stay. You don’t have to go because they’re here. it’s family. As the Aboriginal culture says, everyone’s family. It’s great so even my (non-Indigenous) wife feels comfortable talking with them. When I had the heart attack, they even come up and sat with her while I was in surgery. They didn’t have to do that. Even though me wife had me mother-in-law there, it was like having someone from me own family there, which they are anyway. (P06A)*

While the family member participants had observed the relationality between patients and the BCC team, their own experiences differed. Indeed, they felt excluded from decision making, despite being the person who would carry the caring load when their loved one was discharged. They did not feel informed or empowered, but rather felt bewildered, frightened, and traumatised following their loved ones’ acute coronary event and subsequent hospitalisation.

*It’s been pretty bad. I’d go into the shower, and have a bit of a cry. I didn’t want him to see me upset or anything. Yeah, it’s been really hard. Emotional. (01B family member)*

*So then his brother rang early in the morning, because his sister turned up there upset … and broke down at his house. You know [laughter], it’s had an impact on the whole family …But it’s a big event, you know …. not for him only, for all of us … for all of us. …Well, it would have helped me, well, if somebody would explain to me about his, recovery and what he can do, what he can’t do. (10B family member)*

### Ways of knowing – colonised history, Aboriginal and Torres Strait Islander knowledges

The non-hierarchical nature of the BCC team and the elevation of the HLO and HW role within the the team meant that their combined clinical and cultural knowledges provided a solid foundation for the relationality between the team and the patient-participants (ways of being). Patient-participants’ life stories and lived experiences were situated within a colonised country. Their experiences of the health system in which health authorities have acted in similarly oppressive and discriminatory manner as those from other government institutions impacted their willingness to seek help either before or during their hospital admission. The BCC team understood the historical and cultural context of patient-participants’ concerns and fears, and provided relational care to overcome these barriers, as explored in the following extract.

*The Indigenous culture … we don’t actually ask questions about this and stuff … because there’s fear of, especially at my age, there’s a fear of betrayal in certain different areas in government organisations … You don’t really [trust the government or government institutions] and you grew up with that … and so to see a friendly face (BCC CNC) and to get the information that I wanted, if she didn’t have the answers, she’d be able to get hold of it … (P09A)*

These experiences of ongoing colonisation affected each patient-participant at a profound and individualised level, which was overlain with demeaning and disempowering experiences within the health system. Experiences of family separation and loss due to the ongoing trauma of the Stolen Generations impacted participants’ health and wellbeing [28]. Similarly, everyday experiences of racism, judgement, discrimination, denial of healthcare, and loss and grief due to premature morbidity were common. These experiences negatively impacted participants’ health and wellbeing and contributed to a guarded receptiveness to BCC [29]. For example, one patient-participant recounted having three heart attacks in five years but being denied any medical intervention by the cardiac surgeon. He felt labelled as a smoker and judged as not being worthy of quality, life-saving medical care.

*“…And then when I got down there [transported by helicopter from a regional centre approximately 130km away] … close to dying, on me third heart attack, I again got lectured for five days on chewing gum and patches by the [same] surgeon ... and threatened with not receiving the surgery …we should never ever go to get our health or any – any health help whatsoever and be made feel that we’re not worthy.” (P16A)*

Being ‘*made to feel not worthy*’ was echoed across other participants’ stories of judgement and exclusion in healthcare. For this patient-participant (16A), it was only advocacy from the BCC team, and a change of surgeon, which eventually resulted in him receiving the surgical intervention he needed. For many others, it was the waiting, judgement, and disrespect on general wards which produced the most negative experiences, as expressed by this patient-participant.

*“I felt that they (her young nurses) were impatient, and they were very rude ... they were rude in some of the things that they did, in the comments that they were making about certain patients … while they were in the shower … I may have even said to one of the nurses, “You need to be mindful of what youse are saying when – when people are here” … it was negative, you know.” (P08)*

The BCC team, particularly the HLOs and HWs, supported patient-participants through the contextual and structural challenges they experienced. Health communication was one example where health professionals were accused of using language that no one understood, thus consciously or unconsciously creating a power differential. The BCC HLOs and HWs understood the importance of disrupting these power structures by translating what the doctors’ said into language that the patients understood, highlighted in the following extract.

*“… Some of the things [the doctors said] that I didn’t understand, I’d ask [BCC HLO] to be there … [and I’d ask] what the hell are they talking about? cause doctors think they’re talking to another doctor … I got no idea what you’re talking about …Why go along with all these bloody big names? I don’t care if you went to university and you can read the dictionary backwards, you’ve got to put it in straight English ... not double-dutch that no-one understands. It’s amazing how much that [BCC HLO] knows and can explain what they’re talking about ... so, they support you that way as well. I don’t understand doctors half the time.” (P06A)*

### Ways of doing – holistic care, meeting clients’ diverse needs

This relationality, imbued with their cultural and contextual knowledge, anchored care around the BCC HLOs and HWs, and enabled patients to receive the cardiac care they needed, as their other immediate needs had been addressed. The team took responsibility for the whole person and all their physical, psychological and social needs.

The BCC team knew the patients as unique individuals, were familiar with patient-participants’ life stories and medical histories and were therefore able to provide the necessary supports for each individual patient – it was not a one size fits all approach. The BCC team provided mental health support, companionship, items such as toothbrushes, food, and coffee to increase patient-participants’ comfort while hospitalised, and medical certificates for work, family or justice issues to enable external obligations to be met. The following extract is from a woman who left her job and home in regional Queensland to seek specialist cardiac surgery. The BCC team was stable and supportive throughout the resulting upheavals in her life.

*They’ve been a great support to me. I don’t know how I would have coped without them, to tell you the truth. Because I had so many changes in my life. I went from being full-time worker, independent, to pretty much having everything taken from me … well that’s how I felt, I felt like my world was crumbling ... lost my house, had to quit my job, and was too sick … so they really gave me that mental, emotional support through that ... they followed up with all different [health care] units too, not just my surgeon, or my cardiologist, but with the heart care team or whether it was a physio or anything like that … but if I lost anything [paperwork], I knew I could just call them … just that one point of contact, I didn’t have to worry about being on the phone, (otherwise) I didn’t know who the heck I was calling. (P02A)*

The holistic care provided by the BCC team was not restricted to patient-participants’ hospital admissions but commenced beforehand (for those with a planned admission) and continued after the discharge. For example, the BCC team advocated for elective admissions for people (after 8 years for one patient-participant), sought answers on the wards, and provided extensive post-discharge care involving general practitioners (GPs). Patient-participants appreciated the BCC team’s competence, knowledge, tenacity, and commitment to follow-up. One patient-participant recounted how, post-discharge, she and her GP had repeated teleconference calls with a BCC CNC to titrate heart failure medications.

*“Each fortnight that I go in to see my GP ... we’ve got to ring the PA and talk to the doctor, the cardio or heart specialist. [for a three-way conversation with BCC CNC] … and then he’ll say if we put him up on a higher dosage of an afternoon tablet – it can help my heart muscles more.” (P17A)*

Patient-participants appreciated how the BCC team were cognisant of the reality of their lives, and how the team would advocate for them within the hospital structure. For example, one patient-participant recounted how the BCC team facilitated him receiving a clearance for his commercial drivers’ licence so he could return to work, after the patient had literally ‘chased’ the cardiologist out of the ward to get his questions answered.

*“I had all these questions while I was in there … which I couldn’t get answers to on the doctor’s ward rounds … I followed them … made them take notice of me, you know … I haven’t finished … I needed that answer [about the licence] because I’ve got people depending on me too … and afterwards to get the clearance, for my commercial licence after the stress test, [BCC CNC] … she actually rang, she filed an email through and (the specialist) did get back to me that same day … he rang me up and he said, “Well, you don’t have to wait, you can go back now if you want – your licence is clear now” … Because there’s a real bonus, because they (BCC) were answering questions that I couldn’t get off the doctors. (P09A)*

Patient-participants proudly recalled witnessing the disruption of the hospital power structure, particularly with the BCC HLOs and HWs. This was protective for patient-participants who had experienced disempowerment and racism in healthcare in the past.

*I like how (BCC HLO) carries herself too. I can have a laugh with her, I can have a yarn with her. But she’s very confident. She walks very confident. Like, I’ve seen her say to the nurses before, “call me if [patient name] needed this, this or this, let me know, give me a call and I’ll come up.” So – I don’t know whether she’s had words to them before, or what, but they know to give them a call*. *Actually, I was talking to the nurses in regards to the medical certificate, for my aunt, and I said to one of the nurses, “I’m waiting for this doctor to bring this thing around,” and I sort of explained to her what had happened. And she said, “did you call (BCC HLO)?” And that’s when I said, “You know what, I didn’t even think of that.” And, so I called (BCC nurse) and (BCC HLO), and then it was done straightaway. (P02A)*

### Supporting family members

Whilst patient-participants felt their family members had been well supported by the BCC team throughout their hospital admission, both family member-participants felt they needed more emotional support, better communication, more information during the admission, and more support for the post-discharge rehabilitation period. Both family member-participants recalled the trauma of their loved one suffering an acute coronary event and fear that their loved one may die. One family member-participant recounted being re-traumatised because they were taken to see their loved one in post-operative recovery even though their loved one was still under the effect of the anaesthetic, and to the family member-participant looked as if they were dead. For the other family member-participant, their loved one’s cardiac event triggered deeply traumatic memories of the acute cardiac events and subsequent deaths of other close family members. Recounting the trauma was retraumatising, and there were many tears, both in the stories, and in the story-sharing.

*By the time I got [to the hospital], at the [hospital] reception downstairs, and the reception said to me, “Oh, he’s still in theatre” and then I just broke down ... I just cried and cried … and I think all that feeling like I was holding and trying to hold myself together … and I just freaked out … she even gave me a box of tissues, I was crying, crying, crying … so I was very emotionally threadbare ... and my mind was saying to me, “they cut him open upstairs … now I’m going to see him all cut up, on the side, hooked up to the machine” I saw this vision in my head … [and then] the nurse come and said, “Oh, he’s back in the ward. Come on.” And then [in the ward] I looked and he’s yarning and laughing with the staff ... and I was like, “What!!??” and I’m standing there, I’ve been crying and I was very emotional … (10B family member)*

Communication and handover were other specific areas where family members sought more information and support. As the main caregivers after discharge, both family members were unsure of recovery guidelines and what their loved one was able to do. The followup support reported by patient-participants was not echoed amongst family members.

*“All I was told there by the nurse was, “You have to care for him when he gets home.” … I think it was just an expectation … you’ve got a partner, your partner can look after you … but they didn’t know that the partner was being cared for by the patient … because I’m not the patient … that’s probably why [no one followed up the determine if I was managing] … but you see how emotional I was … what I went through … and I was on my own pain … physically, mentally … everything … I was trying to be strong … I wanted him to see that I can do it ... even though I was struggling (10B family member)*

### Where to from here?

The BCC team recognised the disempowerment and racism experienced by patient-participants during their hospital admission and protected and advocated for participants throughout the duration of the admission. Expanding this influence across the PAH and other hospitals and health care settings was recommended by the patient-participants.

*I think what they got at the PA is gotta be used as a learning curve. And then spread that out and let people know that they’re there. I had to ask [about Aboriginal and Torres Strait Islander liaison officers at another hospital] and I got told they are [there] but there’s only two … Like I said, it’s got to happen more and in a bigger frame than what it is. I’ll even volunteer to go in there and talk to them [hospital administrators]. (P06A)*

## Discussion

The relationality between patients and the BCC team, particularly the Aboriginal and Torres Strait Islander staff who provided a social and cultural anchor for participants, allowed for empowerment and raising of Aboriginal and Torres Strait Islander voice in the hospital structure. The relationality included a responsibility to care for patients as people, not just hearts, and this care extended past clinical boundaries of hospital discharge. The Aboriginal and Torres Strait Islander staff recognised the contextual and structural challenges faced by participants, the disempowerment and interpersonal and institutional racism experienced in healthcare, and allowed the BCC team to protect, advocate and support participants holistically through their cardiac health journeys. Expanding this model of care across the hospital and into broader healthcare spheres, and ensuring compassionate and comprehensive family and caregiver handover, could be areas to expand in the future.

Our analysis framework was informed by theoretical frameworks for Aboriginal and Torres Strait Islander research – Ways of Knowing, Being and Doing [24]. The ways of ‘Being’, the relationality between cardiac patients and the BCC team, was key to supporting and empowering patients within the hospital structure. Littletree et al writes that relationality “allows us to actively participate in our world, ensuring that our interactions are compassionate, loving, and caring, as we become accountable to those with whom we relate.” [30][p.415] This accountability and compassion was evident, extending beyond the clinical boundaries of hospital admission and discharge. This cultural connection enabled patients to speak freely, be themselves, and lessened the distrust engendered by Aboriginal and Torres Strait Islander people’s negative experiences with institutional racism inherent in government institutions. Relationality and relationships between health care providers and consumers has been recognised as a fundamental therapeutic tool in Aboriginal and Torres Strait Islander health research [31–33]. For Aboriginal and Torres Strait Islander people, relationships and connectedness are equally or more important to health and wellbeing than the physical and mechanistic aspects of health care [32, 34, 35].

There is much for non-Indigenous health professionals and researchers to learn from this relationality discourse, and a seeming absence of Aboriginal and Torres Strait Islander or relationality work in the extant medical research literature on rapport [36]. Typically, relationships between health care professionals and health care consumers are spoken of in fraught tones and negative language, such as the boundary ‘violations’ of inappropriate relationships, and the importance of maintaining professionalism [37]. Common to these ‘white medicine’ discourses of health professional-patient relationships is a contractual or extractive nature to the relationship – the professional gives care and expertise, the patient gives a combination of obedience, gratitude and payment. It is within this obedient context that researchers have typically narrowed their gaze to understanding rapport between health provider and health consumer [36]. More attention to the compassion, care, and accountability in Aboriginal and Torres Strait Islander discourses on health care team relationality [30] is overdue.

The relationality within the BCC team and between the BCC team and their patients was demonstrated by the open, transparent and responsive health communications that ensured that patients actually understood their cardiac hospitalisation journey. This contrasts with healthcare more generally, where technical biomedical terminology and jargon is often used despite being incomprehensible to most patients, which demonstrates and reinforces power differentials between health professionals and patients [38]. The BCC HLOs and HWs understood this power disparity and dismantled the language, reducing the power differential. These benefits of elevating Aboriginal and Torres Strait Islander voices and challenging existing power structures is reflected in the discourses on cultural safety [39], health communication[38] and Aboriginal and Torres Strait Islander voice in government [40].

The support offered by BCC HWs and HLOs in navigating these experiences of disempowerment and institutional racism was informed by their knowledge of institutional racism, and their experience of it [41]. Both academia and hospitals are white institutions, with lack of recognition, voice, and support experienced by Aboriginal and Torres Strait Islander health professionals [41]. The empowerment of the BCC HLOs and HWs in relating to other health professionals on cardiology wards, noticed and admired by the patient-participants, has been described in other studies of HWs in discrete specialty teams such as oncology [42]. Embedding and valuing HW staff into discrete, consistent multidisciplinary teams in departments may allow improved status, voice and respect for those workers, with more work exploring HW experiences warranted.

Whilst thorough and timely BCC clinical handover to their GPs was recounted strongly and positively, one part of the holistic treating team which requested more support and handover was to *family*. Whilst numbers were small, family did request more information and to be asked about the carer capabilities at home, rather than staff assuming. It was also illuminating to hear family members express more emotional distress about procedures than patient-participants. Whilst patients must maintain utmost control over privacy, confidentiality, and information flow to family, there is little ethical problem in simply asking patient-family units about discharge needs. Whilst one family member reflected ‘I am not the patient’, they are however the essential care team as soon as the patient leaves hospital. There is a relative paucity of literature on family handover, with carers seeking to be more involved in the discharge process for younger person’s healthcare [43], and in Aboriginal and Torres Strait Islander health a request for family to be more involved in health promotion [44]. Systematic reviews found likely higher carer demands and ill-health for Aboriginal and Torres Strait Islander carers compared to non-Indigenous carers, but there is limited evidence available [45]. Overall involving family in handover appears overlooked in the wider research literature. When reflecting on the definitions of Aboriginal and Torres Strait Islander health [46] and what matters to Aboriginal and Torres Strait Islander adults’ wellbeing [34], family and community wellbeing are essential to patient wellbeing, and should be considered as a natural extension of the holistic treating team. Including family support and handover in a structural fashion is an area where Aboriginal and Torres Strait Islander focussed services could pilot interventions which could positively influence the broader health system.

### Strengths and Limitations

We took great care to attempt to conduct all aspects of this research in a culturally appropriate manner, informed by Aboriginal and Torres Strait Islander research methodologies [24], engaging Aboriginal and Torres Strait Islander research expertise and governance, valuing our story provider’s time, experience, and choices, and recognising the relationships between the BCC team, SE and the story providers. Our findings from southern Queensland regional and urban participants may not translate to other areas of Australia. Despite critical case sampling, we recruited less female patients than male (reflected in the cardiac patient population), less family members, and less rural and remote participants than anticipated, in part due to COVID-19 travel restrictions. To avoid potential bias, we made sure that no member of the BCC team was involved in collecting data and interviewing the patients. The COVID-19 pandemic, and ‘covid-zero’ approach in Australia in 2020, led to halting of recruitment, and when recruitment restarted, restrictions in travel and cautions in access to certain distinct communities or medically vulnerable individuals remained. Telephone interviews were introduced but limitations in relationship development and depth of narrative were noted. Despite critical case sampling, patients with the most social challenges and difficulties during their hospitalisations may have been hard to reach or declined participation. Regardless, whilst every patient’s story was different, the values and reflections shared were more universal amongst participants.

## Conclusion

A key element of the BCC model of care is the employment and empowerment of Aboriginal and Torres Strait Islander staff. These staff were well informed about cardiac disease and were therefore empowered to participate in discussions with health professionals. Their shared cultural knowledge and experiences of ongoing colonisation and institutional racism meant they understood, at a deep and profound level, the concerns, fears and mistrust of the patients towards authoritarian figures, including health professionals. The Aboriginal and Torres Strait Islander staff facilitated the breaking down of those barriers and enabled open communication between patient and doctor. Patients felt cared for, had their needs met, could relax and recover from their acute coronary episode. This core element of the BCC model of care – the caring and relating to patients as humans, rather than a dysfunctional body part - is likely to improve the ability of health services to meet Aboriginal and Torres Strait Islander patients’ needs and produce better outcomes. The wider health system and health academia could benefit from exploring and valuing Aboriginal and Torres Strait Islander discourses of relationality. Family support and handover is an area for all to continue expand efforts. Respecting and valuing our story providers was key to safely sharing these learnings.

## Data Availability

The data generated, analyzed and presented in this study was saved on password protected computers, and not otherwise available publicly, as participants consented to their data being used only for the purposes described in this study.

## List of Abbreviations

PAH: Princess Alexandra Hospital, Brisbane, Australia
BCC: Better Cardiac Care model of care at Princess Alexandra Hospital, Brisbane, Australia
HLO: Aboriginal and Torres Strait Islander Hospital Liaison Officer? Better Cardiac Care
HW: Aboriginal and Torres Strait Islander Health Worker ? Better Cardiac Care
CNC: Clinical nurse consultants (CNCs) ? Better Cardiac Care
RO: Research Officer
SQCoE: Southern Queensland Centre of Excellence in Aboriginal and Torres Strait Islander Primary Health Care
